# Effect of the Most Relevant CYP3A4 and CYP3A5 Polymorphisms on the Pharmacokinetic Parameters of 10 CYP3A Substrates

**DOI:** 10.3390/biomedicines8040094

**Published:** 2020-04-22

**Authors:** Miriam Saiz-Rodríguez, Susana Almenara, Marcos Navares-Gómez, Dolores Ochoa, Manuel Román, Pablo Zubiaur, Dora Koller, María Santos, Gina Mejía, Alberto M. Borobia, Cristina Rodríguez-Antona, Francisco Abad-Santos

**Affiliations:** 1 Clinical Pharmacology Department, Hospital Universitario de La Princesa, Instituto Teófilo Hernando, Universidad Autónoma de Madrid (UAM), Instituto de Investigación Sanitaria La Princesa (IP), 28006 Madrid, Spain; miriam.saiz@salud.madrid.org (M.S.-R.); susanaalmenara@gmail.com (S.A.); marcos.navares@salud.madrid.org (M.N.-G.); mdolores.ochoa@salud.madrid.org (D.O.); manuel.roman@salud.madrid.org (M.R.); pablo.zubiaur@salud.madrid.org (P.Z.); dora.koller@salud.madrid.org (D.K.); ginapaola.mejia@salud.madrid.org (G.M.); 2 Reseach Unit, Fundación Burgos por la Investigación de la Salud, Hospital Universitario de Burgos, 09006 Burgos, Spain; 3 UICEC Hospital Universitario de la Princesa, Plataforma SCReN (Spanish Clinical Reseach Network), Instituto de Investigación Sanitaria la Princesa (IP), 28006 Madrid, Spain; 4 Centro Nacional de Investigaciones Oncológicas (CNIO), 28029 Madrid, Spain; msantosr@cnio.es (M.S.); crodriguez@cnio.es (C.R.-A.); 5 Clinical Pharmacology Department, Hospital Universitario La Paz, 28029 Madrid, Spain; a.borobia@gmail.com; 6 Pharmacology Department, School of Medicine, Universidad Autónoma de Madrid, 28029 Madrid, Spain

**Keywords:** CYP3A4, CYP3A5, pharmacokinetics

## Abstract

Several cytochrome P450 (CYP) CYP3A polymorphisms were associated with reduced enzyme function. We aimed to evaluate the influence of these alleles on the pharmacokinetic parameters (PK) of several CYP3A substrates. We included 251 healthy volunteers who received a single dose of ambrisentan, atorvastatin, imatinib, aripiprazole, fentanyl, amlodipine, donepezil, olanzapine, fesoterodine, or quetiapine. The volunteers were genotyped for *CYP3A4* and *CYP3A5* polymorphisms by qPCR. To compare the PK across studies, measurements were corrected by the mean of each parameter for every drug and were logarithmically transformed. Neither *CYP3A* phenotype nor individual *CYP3A4* or *CYP3A5* polymorphisms were significantly associated with differences in PK. However, regarding the substrates that are exclusively metabolized by CYP3A, we observed a higher normalized AUC (*p* = 0.099) and a tendency of lower normalized Cl (*p* = 0.069) in *CYP3A4* mutated allele carriers what was associated with diminished drug metabolism capacity. *CYP3A4* polymorphisms did not show a pronounced influence on PK of the analysed drugs. If so, their impact could be detectable in a very small percentage of subjects. Although there are few subjects carrying CYP3A4 double mutations, the effect in those might be relevant, especially due to the majority of subjects lacking the CYP3A5 enzyme. In heterozygous subjects, the consequence might be less noticeable due to the high inducible potential of the CYP3A4 enzyme.

## 1. Introduction

Precision medicine uses the tools provided by molecular biology to improve the diagnosis and expand the treatment options of patients based on their biological and molecular profile. Pharmacogenetics, the study of genetic markers that influence the inter-individual variation in drug response, is one of the cornerstones of personalized medicine [1,2]. The role of heritable genetic variation in drug response has been studied since the 1950s [3]. In 1959, Vogel coined the term pharmacogenetics to define a new field that aimed to study the influence of inherited factors on drug response variability through genetic and pharmacological knowledge and methods [4].

Several genes are related to drug response. The U.S. Food and Drug Administration (FDA) describes four gene categories in drug development that can affect the benefit-risk profile of a certain drug: (1) genes relevant to the pharmacokinetics of the drug, (2) genes related to the pharmacological effect of the drug, (3) genes not directly related to the pharmacological effect of the drug, but may predispose to toxicities, and (4) genes that influence the susceptibility or progression of the disease [5]. To date, the most important genes that are related to the pharmacokinetics (PK) of drugs encode cytochrome P450 superfamily (CYP) enzymes.

At least 57 active CYP genes are present in the human genome, along with approximately the same number of pseudogenes. CYP enzymes are divided into 18 families and 44 subfamilies. CYP 1–3 families are particularly active in the detoxification of exogenous chemicals, such as drugs [6]. Every CYP protein sequence is designated as CYP (superfamily root symbol), followed by a number (gene family), then a capital letter (subfamily), and another number (protein). Family members should share >40%, while subfamily members should share >55% amino acid sequence identity [7].

CYP enzymes are responsible for about 75–80% of phase I drug metabolism [8]. Genetic polymorphisms in CYP enzymes which are associated with a characteristic effect in drug metabolism are translated into different metabolizer phenotypes: ultra-rapid metabolizers (UMs), normal metabolizers (NMs), intermediate metabolizers (IMs) and poor metabolizers (PMs). However, these categories give only a static idea about the functionality of an enzyme in an individual. In addition to genetics, variability in CYP activity may be explained by other factors, such as age, sex, morbidity, co-medication, food, or smoking [9].

### 1.1. CYP3A Subfamily

The human CYP3A subfamily consists of 4 isoforms, CYP3A4, CYP3A5, CYP3A7, and CYP3A43 [10]. These isoforms are present in variable proportion in the human liver, being about 30% of the total CYP enzyme content [11]. Genes encoding for *CYP3A* enzymes are located on the 7q21–q22.1 chromosome band [12] along with *CYP3AP1* and *CYP3AP2* pseudogenes, in a cluster of about 200kb [13]. All four CYP3A enzymes comprise 13 exons [14] and show high structural similarity (between 71.5% and 84.1%) [15].

The 5′ untranslated region (UTR) of each human CYP3A gene consists of an average of 101 nucleotides, which is below the mean human 5’-UTR length of 150 nucleotides. On the contrary, the size of the 3′-UTR is highly variable, being 111 nucleotides for CYP3A5, 463 for CYP3A7, 549 for CYP3A43 and 1152 for CYP3A4 [16]. The role of UTRs lies in the regulation of gene expression. Therefore, as CYP3A4 has the longest 3′UTR, it may be greatly regulated [17].

CYP3A43 is generally expressed at low levels in prostate, liver, kidney, and pancreas [10]. It is mainly involved in the metabolism of endogenous compounds, but not drugs [18].

CYP3A7 represents approximately 30–50% of the total CYP450 enzyme amount expressed in fetal liver, where CYP3A4 is not expressed [19]. CYP3A7 was detected also in placenta and endometrium. CYP3A7 is rarely present in adult livers at significant levels as it is gradually substituted by CYP3A4 after birth [18].

**Figure 1:**
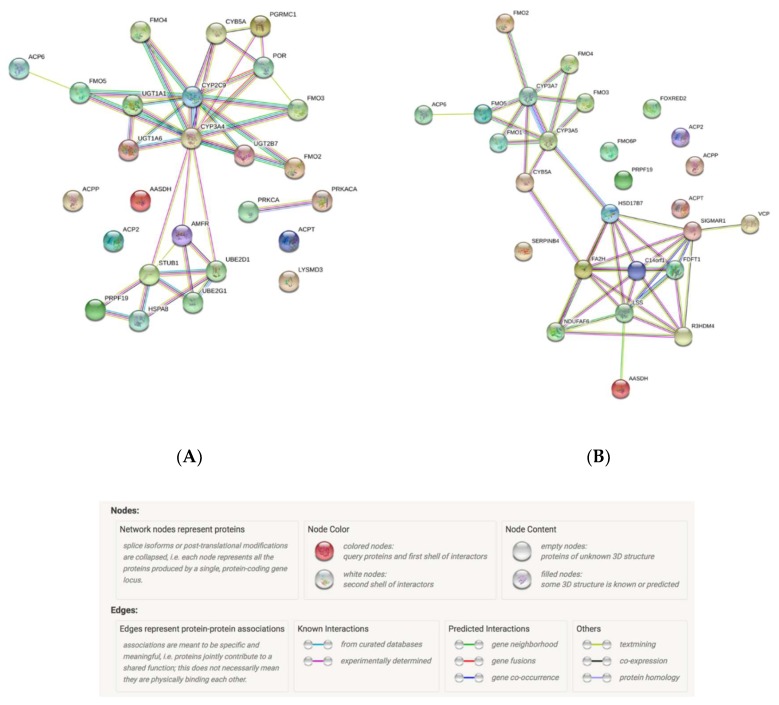
Top CYP3A4 (**A**) and CYP3A5 (**B**) protein interactants. Data obtained from: STRING Interaction Network. Szklarczyk et al. Nucleic acids research 47.D1 (2018): D607-D613.2.

Enzyme activity and functional protein interactions are key to understanding the cellular machinery. In order to fully understand every biological phenomena, it is of importance to study the connectivity of the whole network [20]. CYP3A enzymes are involved in steroid hormone biosynthesis and in lipid and fatty acid metabolism pathways, while interacting with the main molecules described in Figure 1.

### 1.2. CYP3A4 and CYP3A5

Among the human CYP3A enzymes, CYP3A4 and CYP3A5 are considered the most important in drug metabolism [21,22]. Both are highly abundant in the adult liver and intestine [11,23].

In 1984, Kleinbloesem et al. reported a bimodal frequency distribution of the area under the concentration-time curve (AUC) of nifedipine and suggested that a polymorphism was related to its disposition kinetics [24]. Guengerich in 1985 identified and purified the enzyme responsible for the oxidation of nifedipine from rat and human livers, which he named P450 Nifedipine Oxidase. This enzyme is known as CYP3A4 today [25]. Since then, several studies aimed to characterize this enzyme more precisely.

*CYP3A4* gene is located on the 7q22.1 band of the human karyotype [26] with a length of nucleotides [27]. Previously another CYP3A gene, *CYP3A3*, was thought to exist; however, it seems that it is a transcript variant of *CYP3A4*. To date, 8 splice variants encoding different isoforms have been identified. Six of these contain an Open Reading Frame (ORF) and code proteins with the size range between 153 and 534 amino acids [28].

CYP3A4 is expressed predominantly in the liver, where it is the most expressed CYP enzyme [29], ranging from 14.5% to 37% of the hepatic P450 pool [6]. Overall, CYP3A5 is expressed at lower levels than CYP3A4 (~10% of CYP3A4) [30]. Moreover, CYP3A4 is the most abundant CYP enzyme in the human intestinal epithelial cells [31]. Intestinal CYP3A4 plays a role in the bioavailability of some drugs, such as midazolam and cyclosporine via first-pass metabolism during liver transplantation [32,33].

One of the *CYP3A4* most commonly studied reduced-function single nucleotide polymorphism (SNP) is *20, which gives rise to a truncated protein with complete loss of function of the enzymatic activity [34]. Similarly, CYP3A4*22 results in a reduced mRNA expression and a low CYP3A4 enzyme activity [17,35,36].

A previous study characterised CYP3A4 as a highly polymorphic enzyme as CYP3A4*20 allele was present in 1.2% of the Spanish population and up to 3.8% in specific regions [37]. This study described the CYP3A4*20 no function variant with such a high frequency for the first time [37].

Regarding the CYP3A4*22 variant, it is of especial interest to find relevant associations with several drugs, such as tacrolimus [38,39,40,41,42,43]. Briefly, it was linked to lower clearance, and therefore lower dose requirements. Moreover, CYP3A4*22 was also associated with a greater reduction in total and LDL cholesterol levels in an observational study with simvastatin users from a Caucasian cohort [44].

Another relevant variant which was predominantly studied in Caucasians is CYP3A4*1B (rs2740574G), a single base change in the 5’-UTR of *CYP3A4*. At first, this variant was studied related to prostate tumors [45,46]. It was associated with lower enzyme activity and reduced binding of nuclear proteins in vitro [47]. However, other studies contradicted these results [46,48]. In PK studies, CYP3A4*1B was associated to decreased dose-adjusted trough concentrations (C0/D) and higher dose requirements of tacrolimus [38,41,42,49,50,51], and cyclosporine [52,53,54] in transplanted patients. It was also associated with a lower risk of dose decrease or switching therapy during simvastatin treatment [55]. Usually when studied together with CYP3A5*3 or CYP3A4*22, it loses relevance against them [39,41,50]. On the other hand, a significant linkage disequilibrium between this variant and CYP3A5*1 was described, which could partly explain the underlying cause of the observed associations with CYP3A4*1B [56].

CYP3A4*1G variant is located in intron 10 of the *CYP3A4* gene [57] and was predominantly studied in Asian populations [58]. In some studies, CYP3A4*1G carriers showed an increased clearance and a decreased C0/D of tacrolimus [59,60,61,62,63] and cyclosporine treatment [64,65]. CYP3A4*1G was associated with less dose requirements of fentanyl by patient-controlled analgesia (PCA) in the treatment of postsurgical pain [66,67,68,69,70]. However, further evidence is needed from independent studies to confirm these associations.

CYP3A4*2, CYP3A4*12, and CYP3A4*17 alleles have a very low frequency and their functional impact is not established clearly [15,57,71].

Finally, the CYP3A4*3 variant (rs4986910) is a missense polymorphism [71] with very little clinical evidence. In a study with 2735 individuals on statin therapy, CYP3A4*3 was associated with an increase in HDL-cholesterol levels with fluvastatin treatment [72].

To date, eight splice variants of *CYP3A5* have been described. Three of these encode proteins with the size range between 1720 and 4473 amino acids [73]. The presence of the SNP 6986A>G (rs776746) in intron 3 defines CYP3A5*3 and results in a non-functional CYP3A5 protein in homozygous carriers (CYP3A5*3/*3) [74]. This defective variant is the most frequent among Caucasians and Asians. As a result, CYP3A5 is expressed in approximately 10–25% of individuals, depending on their ethnicity (Table 1). When expressed, CYP3A5 can represent about 50% of the total CYP3A hepatic content, which is equal to CYP3A4 activity [75]. On the other hand, CYP3A4 and CYP3A5 usually share substrate specificity. Therefore, a combined phenotype including both enzymes were proposed for several drugs, such as midazolam [76], everolimus [77], statins [78], or tamoxifen [79].

Other variants that result in a non-functional CYP3A5 protein are CYP3A5*6 (rs10264272), which causes an alternative splicing of *CYP3A5* [74] and CYP3A5*7 (rs41303343), an insertion that results in a frameshift [80]. Both are greatly frequent in Latin American and African populations (Table 1). In a study with 140 Latin-American adult kidney transplant recipients under tacrolimus treatment, *CYP3A5* defective haplotypes (*3, *6, *7) showed an increased C0/D [39,40].

Moreover, CYP3A5*8 (rs55817950) and CYP3A5*9 (rs28383479) are defective coding alleles that showed decreased CYP3A5 activity in in vitro models [81]. However, to date, these SNPs have been rarely studied in vivo.

**Figure 2:**
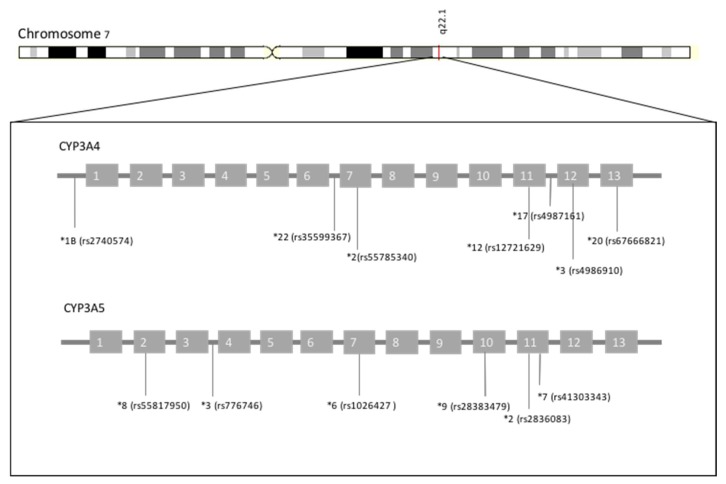
Polymorphic context of CYP3A4 and CYP3A5.

Figure 2 depicts the polymorphic context of *CYP3A4* and *CYP3A5*, with the main polymorphisms described above.

Tacrolimus is the only drug to which CYP3A-genotype guided recommendations are applied. In 2015, the Clinical Pharmacogenetics Implementation Consortium (CPIC) published the guideline for *CYP3A5* genotype and tacrolimus dosing [82]. This association is well established; nevertheless, the variable frequency of CYP3A5*1 allele among different populations (Table 1) makes the utility of the genetic test variable.

In most of the studies that analyzed CYP3A4*22, CYP3A5*3 was also analyzed and in the majority of them the latter showed less associations [83,84]. This could be due to the different contribution of each gene to the metabolism of the different substrates, as it was demonstrated in vitro [85], or can alternatively be caused by a bias related to the low frequency of CYP3A5 expressers in some populations (Table 1).

CYP3A4 protein liver content and activity show an important inter-individual variation ranging from 10 to >100 fold [11,46,86]. In addition, CYP3A4 content was higher in female liver specimens compared to males [29,87]. Furthermore, a higher clearance of various CYP3A substrates was observed in females treated with different drugs, such as nifedipine [88], oxycodone [89], or cyclosporine [90]. CYP3A4 activity may also be affected by the phases of the menstrual cycle [91].

Drugs, herbals or alimentary products can also increase CYP3A4 metabolic activity by increasing enzyme expression. The mechanism of induction involves various nuclear receptors: pregnane X receptor (PXR), constitutive androstane receptor (CAR), glucocorticoid receptor (GR), vitamin D receptor (VDR), and hepatocyte nuclear factor—4 alpha (HNF4α). These nuclear receptors bind to DNA segments present in CYP3A promoter regions, such as PXR responsive element (prPXRE), xenobiotic-responsive enhancer module (XREM), or constitutive liver enhancer module (CLEM4), while increasing transcription and expression of CYP3A4 [6,92]. Some known CYP3A4 inducers are rifampicin, carbamazepine, phenytoin, phenobarbital, paclitaxel, cyclophosphamide, glucocorticoids, and herbs such as St. John’s Wort [92]. CYP3A4 is also susceptible to reversible and irreversible inhibition. Some of the most used drugs that inhibit CYP3A4 are macrolide antibiotics, protease inhibitors, anti-HIV agents, azole antifungals, fluoxetine, verapamil, diltiazem, and several herbal and dietary components [92]. Irreversible or mechanism-based inhibition is more relevant in in vivo drug interactions, specifically, nicotinamide adenine dinucleotide phosphate hydrogen (NADPH)-, time- and concentration-dependent [93].

Evidence of the effect of some CYP3A polymorphisms on the PK of some relevant drugs is scarce and often inconclusive; therefore, its inclusion in prescription guidelines is limited. Our aim was to verify and quantify the effect of the main *CYP3A4* and *CYP3A5* polymorphisms and a merged phenotype on the pharmacokinetic parameters of a large number of CYP3A substrates.

## 2. Materials and Methods

### 2.1. Study Population and Study Design

Our study population comprised 251 healthy volunteers who participated in one of 11 bioequivalence clinical trials (10 of them performed at the Clinical Trials Unit of Hospital Universitario de La Princesa, Madrid, Spain, and 1 at the Clinical Trials Unit of Universidad Autónoma de Madrid, Madrid, Spain) with CYP3A-substrates and agreed to participate in the pharmacogenetic study. The volunteers received a single oral dose of ambrisentan 10 mg (1 trial, *n* = 23), atorvastatin 80 mg (2 trials, *n* = 50), imatinib 400 mg (1 trial, *n* = 12), aripiprazole 10 mg (1 trial, *n* = 26), fentanyl 0.3 mg (1 trial, *n* = 35), amlodipine 10 mg (1 trial, *n* = 25), donepezil 10 mg (1 trial, *n* = 23), olanzapine 5 mg (1 trial, *n* = 25), fesoterodine 8 mg (1 trial, *n* = 13) or quetiapine 25 mg (1 trial, *n* = 19). Each study was a bioequivalence clinical trial in which a test formulation was compared to a reference formulation, after a single oral dose administration under fasting conditions.

All protocols met the terms of the current Spanish Legislation on human clinical research and were approved by the Research Ethics Committee, duly authorized by the Spanish Drugs Agency and under the guidelines of Good Clinical Practice. All participants signed a written informed consent for both the clinical trial and the pharmacogenetic study. Subjects were free to withdraw from the study at any time.

The inclusion criteria for each study were as follows: non-smoking male and female volunteers, age 18–55 years, body mass index within the 18.5–30.0 range, free from any organic or psychiatric conditions, with normal vital signs, electrocardiogram, medical records and physical examination.

Healthy volunteers were asked prior to study inclusion if they had taken any medications or supplements in the past month. Those volunteers who had taken any drugs or supplements were excluded from the clinical trial. Concomitant pharmacological treatments were not administered during none of the phases of the study. At the time of admission, the night prior to the administration of the study drug, volunteers were again asked if they had taken any concomitant medication during the time they had not been admitted to the Clinical Trials Unit.

### 2.2. Pharmacokinetic Analysis

Since the determination of every drug plasma concentration was outsourced, all samples were stored at −20 ± 5 °C or −75 ± 5 °C until their shipment to the external analytical laboratory. Each quantification was accomplished by a high-performance liquid chromatography coupled to tandem mass spectrometry (HPLC-MS/MS) validated method, according to the European Medicines Agency guidelines. Pharmacokinetic parameters were calculated by means of a non-compartmental analysis with WinNonlin Professional software, Version 2.0 (Pharsight Corporation, Palo Alto, California), as described previously [94]. The AUC and the maximum concentration (C_max_) were corrected by dose/weight. Clearance (Cl) and volume of distribution (Vd) were divided by weight.

### 2.3. Genotyping

DNA was obtained from 1 mL of peripheral blood samples using MagNA Pure LC DNA Isolation Kit in an automatic DNA extractor (MagNa Pure^®^ System, Roche Applied Science, Indianapolis, Indiana). CYP3A4*2 (rs55785340), CYP3A4*3 (rs4986910), CYP3A4*12 (rs12721629), CYP3A4*17 (rs4987161) and CYP3A4*22 (rs35599367) and CYP3A5*2 (rs28365083), CYP3A5*3(rs776746), CYP3A5*6 (rs10264272), CYP3A5*7 (rs41303343), CYP3A5*8 (rs55817950), and CYP3A5*9 (rs28383479) polymorphisms were genotyped by quantitative PCR (qPCR), in a QuantStudio 12k Flex instrument (Applied Biosystems, Foster City, CA, USA). We analysed these SNPs as they were the ones included in the commercial and predesigned TaqMan™ OpenArray™ PGx Express Panel (Applied Biosystems, Foster City, CA, USA).

The CYP3A4*20 (rs67666821) polymorphism call was assessed by KASPar SNP Genotyping System (LGC Genomics, Herts, UK) in an ABI PRISM 7900HT Sequence Detection System (Applied Biosystems, Darmstadt, Germany). All CYP3A4*20 carriers were confirmed by Sanger sequencing in an ABI PRISM 3700 DNA Analyser capillary sequencer (Applied Biosystems, Foster City, California, USA) [37].

The *1 allele, considered as wild-type, was assigned when the subject lacked all of the analyzed alleles.

### 2.4. Statistical Analysis

The influence of *CYP3A4* and *CYP3A5* genotypes were analysed separately. However, to assess the influence of both enzyme conjunctively, *CYP3A4* and *CYP3A5* genotypes were merged into a “CYP3A phenotype” as follows: subjects with at least one *CYP3A4* reduced activity allele (i.e., *CYP3A4* *1/*22, *1/*3, *22/*22 or *3/*22) and no CYP3A5 activity (*CYP3A5* *3/*3,*3/*6 or *3/*7) were considered PM; subjects with normal CYP3A4 activity (*CYP3A4* *1/*1) and no CYP3A5 activity (*CYP3A5* *3/*3, *3/*6 or *3/*7) were considered IM; finally, subjects with normal CYP3A4 activity (CYP3A4*1/*1) and at least one *CYP3A5* functional allele (*CYP3A5* *1/*1 or *1/*3) were categorized as extensive metabolizers (EM), based on Sanchez Spitman et al. [79]

Statistical analyses were accomplished with the SPSS 22.0 software (SPSS Inc., Chicago, Illinois, USA). *p* values < 0.05 were considered statistically significant. Chi-square test was used to define sex-related differences in the genotypic frequencies. T-test or ANOVA were used to evaluate differences in PK among sexes and genotypes. To compare the PK parameters across studies, they were divided by the mean of each parameter for every drug and logarithmically transformed, thus termed as normalized parameter. The power analysis to know how many patients would be necessary to be evaluated in order to reveal a difference in PK parameters was performed with the G.power 3.1.9.7 software.

## 3. Results and Discussion

From all the volunteers included, 164 (65.3%) were Caucasians, 81 (32.3%) were Latin-Americans, four (1.6%) were Blacks, and two (0.8%) were Arabs. Regarding sex, 102 (40.6%) were women, while 149 (59.4%) were men. Mean age was 27.8 years old (ranging between 18 and 55). Mean weight was 69.6 kg (ranging between 44.4 and 98.5 kg) and mean height was 1.70 m (ranging between 1.49 and 1.97 m). Men were younger (26.5 ± 6.5 years vs. 29.8 ± 9.3 years), weighted more (76.0 ± 9.1 kg vs. 60.2 ± 8.1 kg) and were taller (1.76 ± 0.06 m vs. 1.61± 0.06 m) than women, *p* < 0.001.

Mean age was 25.8 ± 6.0, 32.0 ± 9.8, 32.0 ± 9.1 and 23.0 ± 0.0 years old (*p* < 0.001) in Caucasians, Latin-Americans, Blacks, and Arabs, respectively. Mean weight was 69.0 ± 11.3, 70.1 ± 12.1, 85.7 ± 5.2, 63.6 ± 17.0 kg (*p* = 0.033) in Caucasians, Latin-Americans, Blacks, and Arabs, respectively. Finally, mean height was 1.72 ± 0.08, 1.67 ± 0.11, 1.74 ± 0.06 and 1.70 ± 0.13 m (*p* = 0.001) in Caucasians, Latin-Americans, Blacks, and Arabs, respectively.

Table 2 shows the main *CYP3A* genotypes found in our study. None of the genotyped subjects carried CYP3A4*2, CYP3A4*12, CYP3A4*17, CYP3A5*2, CYP3A5*8, or CYP3A5*9 alleles. Our allele frequencies (AF) did not differ from those reported in the literature: CYP3A4*3 AF was 0.3% (Minor allele frequency, MAF: <1%) [95], CYP3A4*20 AF was 0.9% (MAF: <1%) [96], and CYP3A4*22 AF was 2.3% (Iberian MAF: 3.7%; Latin MAF: 2.6%) [97]. Additionally, CYP3A5*3 AF was 87.2% (Iberian MAF: 92.5%; Latin MAF: 79.7%) [98], CYP3A5*6 AF was 1.5% (Iberian MAF: 0.9%; Latin MAF: 2.3%) [99] and CYP3A5*7 AF was 0.2% (MAF: <1%) [100]. No statistically significant difference was found in *CYP3A* genotype frequencies among sexes.

Sex influenced the PK parameters since women showed higher normalized T_1/2_ (1.05 ± 0.34 vs. 0.96 ± 0.30, *p* = 0.03) and normalized Vd (1.06 ± 0.41 vs. 0.96 ± 0.32, *p* = 0.057), compared to men (Table 3). No statistically significant difference was found between men and women in normalized AUC, C_max_, Cl and T_max_. A higher T_1/2_ in women is the opposite of what is expected according to the greater CYP3A4 content in women [29,87]. The higher Vd could be due to the higher fat percentage in women. However, these differences are minor and possibly not sufficient to establish a dose adjustment based on sex.

Regarding the influence of CYP3A alleles or phenotype categories on normalized PK parameters, we performed a separate analysis for each compound (Supplementary Table S1) that showed no significant association of CYP3A polymorphisms for ambrisentan, aripiprazole, donepezil, atorvastatin and olanzapine. However, some associations were found for amlodipine (higher AUC and lower Cl in CYP3A5 PM), imatinib, and quetiapine (higher AUC in CYP3A4 mutated allele carriers). Although not significant, some tendencies were observed in CYP3A4 mutated alleles carriers for fentanyl (higher AUC and lower Cl) and fesoterodine (lower AUC and higher Cl). However, the limited number of individuals carrying mutations in each compound separately makes it difficult to reach any conclusion. Therefore, we merged the compounds for a conjunct analysis.

Regarding the influence of CYP3A genotypes on the PK parameters of the analysed compounds, we observed that neither CYP3A phenotype nor individual *CYP3A4* or *CYP3A5* polymorphisms were significantly associated with PK variability (Table 2). This fact could be explained by the influence of other metabolizing enzymes (i.e., CYP2D6 for aripiprazole [101]), whose polymorphisms might have a higher impact on PK parameters of some drugs.

Although it was not statistically significant, when analysing the substrates that are only metabolized by CYP3A (ambrisentan, atorvastatin, imatinib, fentanyl, amlodipine and quetiapine), some tendencies could be observed (Table 4). Subjects with *CYP3A4* mutated alleles (*3, *20, *22) showed higher normalized AUC (*p* = 0.099) and almost significantly lower normalized Cl (*p* = 0.069), which could be a result of a diminished metabolism capacity. Moreover, the only subject carrying double mutation (CYP3A4*3/*22) had a 53% increase in AUC and a 53% lower Cl. However, the validity of this result need to be confirmed in other studies due to the limited sample size.

In a previous study with the CYP3A-substrate fentanyl, our group was able to demonstrate that carrying CYP3A4*22 polymorphism results in higher AUC and lower Cl [102]. Here, congruently, the same tendency was observed, yet the significance level was not reached as we used AUCt instead of AUCinf [102]. Moreover, the validity of our previous results need to be confirmed in other studies with similar settings due to the small sample size. Other *CYP3A4* genotypes were not related to different exposure to fentanyl [103]. Regarding *CYP3A5*, the presence of *3 allele was linked to a two-fold increase in fentanyl systemic exposure [104]. However, we could not find any homozygous wild-type subject to compare with CYP3A5*3/*3 carriers.

CYP3A4*22 and CYP3A5*3 were not associated with lipid reductions in 105 children and adolescents with familial hypercholesterolemia treated with atorvastatin [105]. Moreover, CYP3A4*22 neither affected the lipid reduction response to atorvastatin and simvastatin treatment in 416 adults [106]. However, Rosales et al. described a better response to atorvastatin in Chilean patients who carried CYP3A4*1B [107]. Likewise, Peng et al. found an influence of the polymorphism CYP3A4*1G (rs2242480) on atorvastatin treatment in patients with ischemic stroke [108]. However, these two polymorphisms were not analysed in our study since they were not included in the commercial TaqMan™ OpenArray™ PGx Express Panel (Applied Biosystems, Foster City, CA, USA).

Indeed, no association with CYP3A4*1G was reported in tacrolimus concentrations in paediatric patients [109] or imatinib pharmacokinetics in Chinese patients [110]. Another *CYP3A4* polymorphism, rs755828176, was significantly associated with dose-adjusted trough plasma concentrations of imatinib and its metabolite [111]. Nevertheless, this frameshift variant is reported not to be present in our population [112]. Another study in 112 patients with chronic myeloid leukaemia found no relationship between *CYP3A4* rs2740574 and CYP3A5*3 and imatinib plasma levels and therapeutic response [113]. Neither did another study in 82 patients treated with imatinib [114]. Indeed, Petain et al. stated that morphologic and biological characteristics have a greater effect on imatinib PK than pharmacogenetics [115]. However, CYP3A5*3 was associated with higher imatinib trough plasma concentrations in Nigerian population [116] and a significant lower risk of acquiring imatinib resistance, while CYP3A4*18 had no statistically significant effect [117]. In our study, the only subject with the CYP3A4*20 allele showed higher imatinib levels (*p* = 0.046), although it is not conclusive due to the limited sample size in our study; the frequency of CYP3A4*20 previously found in Spanish population (1.2%) was not reached [37]. Moreover, in contrast to the study by Adeagbo et al. [116], CYP3A5 PMs showed lower AUC compared to the only IM subject, although, as before, it is not conclusive.

According to our knowledge, there is no study evaluating the effect of polymorphisms in CYP3A on the PK of ambrisentan. However, in our study, it does not appear that *CYP3A4* or *CYP3A5* polymorphisms influence its elimination.

Regarding amlodipine, Huang et al. described an altered antihypertensive efficacy of amlodipine related to CYP3A4*1G and CYP3A5*3 alleles in patients with hypertension following renal transplantation [118]. Moreover, in a study in 40 healthy volunteers, CYP3A5*3/*3 carriers exhibited lower plasma amlodipine concentrations [119]. In our study, although not significant, we found the lowest amlodipine plasma concentration in the only *CYP3A5* *1/*1 subject. Indeed, we found a significantly higher Cl in this subject, but our sample size is not sufficient to arise any conclusion.

Regarding quetiapine, as shown in Supplementary Table S1, we found significant higher plasma levels in carriers of a *CYP3A4* mutated allele (AUC 1107.7 ± 376.1 ng·h·Kg/mL·mg vs. 588.9 ± 272.6 ng·h·Kg/mL·mg in wild-type subjects, *p* = 0.024). A previous work from our laboratory showed that quetiapine AUC was influenced by polymorphisms in *CYP1A2* [120]. According to our knowledge, a direct relationship between *CYP3A4* polymorphisms and quetiapine was not described before. Notwithstanding, the association we found has to be interpreted with caution, given that only two subjects were carriers of mutations. Moreover, Shilbayeh et al. described in a population PK model a 29% increased Cl of quetiapine in *CYP3A5* *1/*1 subjects compared to*1/*3 and *3/*3, but they stated that these simulations do not call for a genotype-based quetiapine dosing scheme [121]. Indeed, polymorphisms in *CYP3A5* had no significant influence on steady-state serum concentrations of quetiapine in psychiatric patients [122]. In our study, we found no difference in any PK parameter of quetiapine according to *CYP3A5* genotypes.

Finally, in regards to the other substrates that are not only metabolized by CYP3A, Noetzli et al. evaluated the effect of polymorphisms in *CYP2D6* and CYP3A, finding that only CYP2D6 influenced donepezil clearance [123]. Moreover, another study in 54 patients did not find any statistically significant association between *CYP3A4* or *CYP3A5* genotypes and plasma donepezil concentrations or clinical response [124].

Moreover, in the current work, when analysing the combined effect of *CYP3A4* and *CYP3A5* into the CYP3A phenotype, the tendencies towards higher AUC and lower Cl disappeared. It is of great importance to increase our sample size and to analyse whether the influence of *CYP3A4* polymorphisms alone is sufficient to have a clear effect on CYP3A-substrates PK parameters, since the majority of subjects lack CYP3A5 activity. With the 251 subjects included in the study the statistical power achieved was 0.81 for the ANOVA analysis of CYP3A phenotype and for an expected effect size of 0.20 for all the normalized PK parameters. The effect size observed was 0.074 for normalized AUC, 0.045 for normalized C_max_, 0.090 for normalized T_1/2_, 0.074 for normalized T_max_, 0.081 for normalized Cl, and 0.063 for normalized Vd. The sample size required for a smaller effect size of 0.09 (if clinically relevant) in an ANOVA analysis of normalized PK variables is 1.194.

Moreover, further approaches might shed light on this study field, such as the design of human recombinant microsomes with different *CYP3A4* and *CYP3A5* polymorphisms, which might allow the assessment of these polymorphisms on in vitro PK, and its comparison with the in vivo PK data. For instance, the study of recombinant CYP3A human liver microsomes showed that CYP3A5 expression influenced the metabolism of the antiviral asunaprevir, but not daclatasvir and beclabuvir [125].

Finally, it is important to note that CYP3A4 is an enzyme that is easily induced and inhibited by a wide variety of drugs, so the effect of polymorphisms, if any, could be masked in patients with multiple medications. Since this enzyme metabolizes many of the drugs actually on the market, it is likely that several CYP3A4 substrates are used concomitantly and that some of them are also CYP3A4 inhibitors or inducers of CYP3A4.

### Study Limitations

Our study was performed after a single-dose administration of each drug to healthy volunteers, that does not allow the assessment of long-term effectiveness and safety. Our results might differ from patients under chronic treatment. Nevertheless, this study design allows the investigation of genetic polymorphisms affecting PK while avoiding the influence of other confounding factors, such as smoking or concomitant treatment. However, further research is needed to increase the statistical power of these results, especially to evaluate the influence in homozygote subjects for inactivating alleles. Finally, there is a possibility that other polymorphisms that were not included in the study could be functionally impactful, but we did not screen for them.

## 4. Conclusions

Our main conclusion is that *CYP3A* polymorphisms themselves do not have a pronounced influence on the PK parameters of the CYP3A-substrates analysed in this study. If so, the influence of *CYP3A4* polymorphisms could be detectable in a very small percentage of subjects, so the evidence so far is scarce and does not support a dose adjustment in the majority of CYP3A-metabolized drugs. Indeed, it is of importance to highlight that, although there are few subjects carrying *CYP3A4* double mutations, the effect in those might be relevant. The fact that the majority of subjects lack the CYP3A5 enzyme, might make the effect of *CYP3A4* double mutations even more important when present. However, in *CYP3A4* heterozygous subjects the consequence might be less noticeable, partly due to the high inducible potential of CYP3A4 enzyme.

## Figures and Tables

**Table 1:** Allele frequencies of the most studied variants of CYP3A4 and CYP3A5.

CYP3A Allele	Reference SNP Identifier	In Vitro Effect	PK Effect in CYP3A Metabolism	Minor Allele Frequencies			
				Europeans	Latin-Americans	Africans	East Asians
CYP3A4*22	rs35599367	↓ [35,36]	↓	50/1006 (4.97%)	18/694 (2.59%)	1/1322 (0.08%)	0/1008 (0%)
CYP3A4*1B	rs2740574	(= [46], ↓ [47]?, ↑ [48]?)	(↑?) [38], [41,42,49,50,51,52,53,54]	28/1006 (2.78%)	73/694 (10.52%)	1012/1322 (76.55%)	4/1008 (0.4%)
CYP3A4*1G	rs2242480		(↑?) [59], [60,61,62,63,64,65]	82/1006 (8.15%)	273/694 (39.34%)	1124/1322 (85.02%)	270/1008 (26.79%)
CYP3A4*3	rs4986910		(↓?) [72]	7/1006 (0.70%)	5/694 (0.72%)	1/1322 (0.08%)	0/1008 (0%)
CYP3A4*20	rs67666821	None	↓	26/64600 (0.04%)~	22/13290 (0.17%)~	3/42021 (0.01%)~	0/3134 (0.00%)~
CYP3A5*3	rs776746	↓	↓	949/1006 (94.33%)	553/694 (79.68%)	238/1322 (18.00%)	719/1008 (71.33%)
CYP3A5*6	rs10264272	(↓?) [74]	(↓?) [39], [40]	3/1006 (0.30%)	16/694 (2.31%)	204/1322 (15.43%)	0/1008 (0.00%)
CYP3A5*7	rs41303343	=/↓	(↓?) [39], [40]	0/942 (0.00%)^	27/1090 (2.48%)^	174/2014 (8.64%)^	0/480 (0.00%)^
CYP3A5*8	rs55817950	↓		0/ 113648 (0.00%)	0/ 34590 (0.00%)	0/ 16216 (0.00%)	0/ 18391 (0.00%)
CYP3A5*9	rs28383479	↓		0/113434(0.00%)	0/34340(0.00%)	0/16226(0.00%)	2/18358 (0.01%)

Minor allele frequencies obtained from Ensemble Genome Browser. PK effect in CYP3A metabolism obtained from PharmVar or cited studies. ~ Data from gnomAD browser v3. ^Data from CPIC guideline for Tacrolimus and CYP3A5: https://cpicpgx.org/guidelines/guideline-for-tacrolimus-and-cyp3a5/. ? Not enough evidence.

**Table 2:** CYP3A genotype and phenotype frequencies in our study population.

**Gene**	**Genotype**	**N**	**%**
CYP3A4	*1/*1	233	92.8
	*1/*20	5	2.0
	*1/*22	11	4.4
	*1/*3	1	0.4
	*3/*22	1	0.4
CYP3A5	*1/*1	5	2.0
	*1/*3	45	17.9
	*3/*3	192	76.5
	*3/*6	8	3.2
	*3/*7	1	0.4
	**Phenotype**	**N**	**%**
CYP3A	PM	17	6.8
	IM	184	73.3
	EM	50	19.9

Abbreviation: CYP, cytochrome p450 oxidase.

**Table 3:** Pharmacokinetic parameters (normalized by the mean of each drug) according to CYP3A genotypes and phenotype and sex in all the included drugs.

Gene	Genotype/Phenotype	Pharmacokinetic Parameter					
		Normalized AUC	Normalized C_max_	Normalized T_1/2_	Normalized T_max_	Normalized Cl	Normalized Vd
CYP3A4	*1/*1 (*n* = 233)	0.99 (0.35)	0.99 (0.42)	0.99 (0.32)	1.00 (0.56)	0.99 (0.38)	1.00 (0.37)
	*1/*20 (*n* = 5)	1.12 (0.24)	0.96 (0.33)	1.18 (0.24)	0.66 (0.27)	0.73 (0.18)	0.90 (0.10)
	*1/*22 (*n* = 11)	1.06 (0.46)	0.98 (0.38)	0.99 (0.18)	1.00 (0.71)	0.96 (0.43)	0.98 (0.43)
	*1/*3 + *3/*22 (*n* = 2)	1.24 (0.40)	1.25 (0.10)	1.07 (0.28)	0.93 (0.03)	0.75 (0.39)	0.83 (0.18)
	*p*-value	0.523	0.738	0.566	0.482	0.338	0.874
CYP3A4	Wild-type (*n* = 233)	0.99 (0.35)	0.99 (0.42)	0.99 (0.32)	1.00 (0.56)	0.99 (0.37)	1.00 (0.37)
	Mutated (*n* = 18)	1.10 (0.39)	1.00 (0.34)	1.05 (0.20)	0.90 (0.58)	0.87 (0.37)	0.94 (0.34)
	*p*-value	0.240	0.853	0.276	0.351	0.146	0.428
CYP3A5	*1/*1 (*n* = 5)	0.90 (0.30)	1.13 (0.48)	0.88 (0.19)	0.73 (0.26)	1.20 (0.51)	1.05 (0.27)
	*1/*3 (*n* = 45)	1.02 (0.32)	1.02 (0.44)	1.04 (0.36)	0.90 (0.52)	0.94 (0.38)	0.96 (0.23)
	*3/*3 + *3/*6 + *3/*7 (*n* = 201)	0.99 (0.37)	0.99 (0.40)	0.99 (0.31)	1.03 (0.57)	0.98 (0.38)	1.00 (0.39)
	*p*-value	0.710	0.626	0.731	0.257	0.486	0.833
CYP3A	EM (*n* = 50)	1.01 (0.31)	1.03 (0.44)	1.02 (0.35)	0.88 (0.50)	0.97 (0.39)	0.97 (0.23)
	IM (*n* = 183)	0.99 (0.37)	0.99 (0.41)	0.98 (0.31)	1.03 (0.57)	0.99 (0.37)	1.01 (0.40)
	PM (*n* = 18)	1.09 (0.39)	1.00 (0.34)	1.05 (0.21)	0.93 (0.59)	0.87 (0.37)	0.94 (0.34)
	*p*-value	0.408	0.692	0.501	0.155	0.324	0.720
Sex	Men (*n* = 149)	1.00 (0.37)	1.00 (0.43)	0.96 (0.30)	0.99 (0.58)	0.99 (0.38)	0.96 (0.32)
	Women (*n* = 102)	0.99 (0.35)	1.00 (0.39)	1.05 (0.34)	1.01 (0.54)	0.97 (0.37)	1.06 (0.41)
	*p*-value	0.765	0.832	0.030	0.426	0.889	0.057

Abbreviation: AUC, area under the curve; C_max_, maximum plasma concentration; T_max,_ time to reach the maximum plasma concentration; T_1/2_, half-life; Cl, total drug clearance adjusted for bioavailability; Vd, volume of distribution adjusted for bioavailability; CYP, cytochrome p450 oxidase.

**Table 4:** Pharmacokinetic parameters (normalized by the mean of each drug) according to CYP3A genotypes and phenotype and sex in pure CYP3A-substrates (ambisentran, atorvastatine, imatinib, fentanyl, amlodipine and quetiapine).

Gene	Genotype/Phenotype	Pharmacokinetic Parameter					
		Normalized AUC	Normalized C_max_	Normalized T_1/2_	Normalized T_max_	Normalized Cl	Normalized Vd
CYP3A4	*1/*1 (*n* = 150)	0.98 (0.41)	1.00 (0.49)	1.00 (0.31)	1.00 (0.56)	1.00 (0.40)	1.00 (0.41)
	*1/*20 (*n* = 4)	1.08 (0.26)	0.91 (0.36)	1.13 (0.25)	0.74 (0.24)	0.78 (0.16)	0.89 (0.12)
	*1/*22 (*n* = 9)	1.14 (0.46)	1.02 (0.39)	1.01 (0.19)	1.01 (0.80)	0.85 (0.39)	0.91 (0.45)
	*3/*22 (*n* = 1)	1.53	1.32	1.27	0.96	0.47	0.71
	*p*-value	0.317	0.796	0.626	0.822	0.194	0.673
CYP3A4	Wild-type (*n* = 150)	0.98 (0.41)	1.00 (0.49)	0.99 (0.31)	1.00 (0.56)	1.00 (0.40)	1.00 (0.41)
	Mutated (*n* = 14)	1.15 (0.40)	1.01 (0.37)	1.06 (0.20)	0.93 (0.64)	0.81 (0.33)	0.89 (0.36)
	*p*-value	0.099	0.723	0.274	0.459	0.069	0.264
CYP3A5	*1/*1 (*n* = 2)	0.96 (0.49)	1.24 (0.72)	0.83 (0.19)	0.75 (0.48)	1.25 (0.81)	0.99 (0.47)
	*1/*3 (*n* = 23)	1.02 (0.42)	1.01 (0.59)	1.03 (0.40)	0.94 (0.63)	0.97 (0.48)	0.92 (0.28)
	*3/*3 + *3/*6 + *3/*7 (*n* = 139)	1.00 (0.41)	0.99 (0.45)	1.00 (0.29)	1.01 (0.56)	0.98 (0.38)	1.01 (0.42)
	*p*-value	0.987	0.760	0.834	0.538	0.731	0.818
CYP3A	EM (*n* = 25)	1.01 (0.42)	1.03 (0.59)	1.01 (0.39)	0.92 (0.62)	0.99 (0.49)	0.93 (0.29)
	IM (*n* = 126)	0.98 (0.41)	1.00 (0.47)	0.99 (0.30)	1.01 (0.55)	0.99 (0.38)	1.02 (0.43)
	PM (*n* = 13)	1.14 (0.41)	0.94 (0.28)	1.06 (0.21)	0.97 (0.65)	0.82 (0.34)	0.91 (0.37)
	*p*-value	0.342	0.988	0.566	0.560	0.280	0.526
Sex	Men (*n* = 95)	0.99 (0.43)	0.98 (0.50)	0.96 (0.27)	0.96 (0.57)	1.00 (0.41)	0.96 (0.34)
	Women (*n* = 69)	1.01 (0.39)	1.03 (0.44)	1.05 (0.34)	1.04 (0.56)	0.96 (0.38)	1.05 (0.48)
	*p*-value	0.747	0.278	0.093	0.209	0.618	0.304

Abbreviation: AUC, area under the curve; C_max_, maximum plasma concentration; T_max_, time to reach the maximum plasma concentration; T_1/2_, half-life; Cl, total drug clearance adjusted for bioavailability; Vd, volume of distribution adjusted for bioavailability; CYP, cytochrome p450 oxidase.

## Supplementary Materials

Supplementary materials can be found at https://www.mdpi.com/2227-9059/8/4/94/s1.
