# Supplementary material for: Effect of the Most Relevant CYP3A4 and CYP3A5 Polymorphisms on the Pharmacokinetic Parameters of 10 CYP3A Substrates
Source: Biomedicines. 2020 Apr 22;8(4):94. doi: 10.3390/biomedicines8040094 (PMC7235792; doi:10.3390/biomedicines8040094)
Supplement: Supplementary file 1 [file biomedicines-08-00094-s001.pdf]

Supplementary table S1. Pharmacokinetic parameters according to CYP3A genotypes and phenotype in every individual drug included.

**Ambrisentan**							
Gene	Genotype/phenotype	AUC/dW (ng·h·Kg/mL·mg)	C_{max}/dW (ng·kg/mL·mg)	$T_{1/2}$ (h)	T_{max}(h)	Cl (L/h·kg)	Vd (L/kg)
CYP3A4	Wild-type (n=22)	37059.3 (9472.6)	5295.7 (832.2)	11.8 (4.4)	1.8 (1.0)	0.03 (0.01)	0.43 (0.13)
	Mutated (n=1)	36619.2	5325.6	16.1	1.5	0.02	0.45
	p-value	0.964	0.972	0.351	0.772	0.447	0.837
CYP3A5	IM (n=1)	45314.2	4754.0	15.8	1.5	0.02	0.45
	PM (n=22)	36664.1 (9291.5)	5321.6 (823.3)	11.8 (4.3)	1.8 (1.0)	0.03 (0.01)	0.43 (0.13)
	p-value	0.373	0.508	0.379	0.772	0.454	0.860

Amlodipine							
Gene	Genotype/phenotype	AUC/dW (pg·h·Kg/mL·mg)	C_{max}/dW (pg·Kg/mL·mg)	$T_{1/2}$ (h)	T_{max}(h)	Cl (L/h·kg)	Vd (L/kg)
CYP3A4	Wild-type (n=23)	1909348.9 (377107.5)	58670.3 (10990.6)	34.6 (7.0)	5.5 (1.8)	0.41 (0.11)	19.9 (3.0)
	Mutated (n=2)	2123474.6 (91340.7)	60134.8 (5097.6)	40.3 (10.5)	5.0 (1.4)	0.33 (0.01)	19.4 (4.7)
	p-value	0.440	0.856	0.296	0.707	0.351	0.846
CYP3A5	NM (n=1)	1175122.2	43408.7	24.5	6.0	0.74	26.4
	IM (n=5)	2052800.7 (284277.7)	55850.7 (10584.0)	38.5 (8.1)	6.6 (2.1)	0.36(0.09)	19.2 (2.2)
	PM (n=19)	1932781.4 (353788.7)	60369.7 (10306.7)	34.8 (6.7)	5.1 (1.7)	0.40 (0.09)	19.7 (2.9)
	p-value	0.086	0.240	0.197	0.291	0.003	0.079

Aripiprazole							
Gene	Genotype/phenotype	AUC/dW (ng·h·Kg/mL·mg)	C_{max}/dW(ng·kg/mL·mg)	$T_{1/2}$ (h)	T_{max}(h)	Cl (mL/h·kg)	Vd (mL·kg)
CYP3A4	Wild-type (n=25)	10148.9 (2535.4)	326.5 (95.3)	56.3 (23.9)	3.0 (2.3)	0.07 (0.03)	4.6 (0.9)
	Mutated (n=1)	13098.4	393.6	79.0	1.0	0.04	4.3
	p-value	0.265	0.497	0.362	0.413	0.332	0.751
CYP3A5	NM (n=1)	11792.4	498.1	65.9	2.0	0.04	4.4

	IM (n=4)	10603.1 (1550.0)	377.4 (68.0)	58.5 (23.6)	1.5 (0.4)	0.06 (0.02)	4.5 (0.2)
	PM (n=21)	10124.6 (2758.9)	311.8 (90.7)	56.5 (24.9)	3.2 (2.4)	0.07 (0.03)	4.6 (1.0)
	p-value	0.795	0.078	0.928	0.386	0.704	0.954

Donepezil							
Gene	Genotype/phenotype	AUC/dW (pg·h·Kg/mL·mg)	C_{max}/dW (pg·Kg/mL·mg)	$T_{1/2}$ (h)	T_{max}(h)	Cl (L/h·kg)	Vd (L/kg)
CYP3A4	Wild-type (n=21)	3045037.2 (531336.9)	126941.9 (30473.0)	44.0 (16.0)	2.2 (1.0)	0.24 (0.06)	14.0 (2.6)
	Mutated (n=2)	2717340.1 (246516.0)	141017.6 (14280.3)	38.3 (0.4)	2.0 (0.0)	0.27 (0.03)	14.9 (1.8)
	p-value	0.405	0.532	0.631	0.762	0.503	0.631
CYP3A5	NM (n=1)	2105891.8	82787.2	35.8	2.0	0.35	18.0
	IM (n=6)	3171300.6 (609051.7)	141307.2 (27436.9)	51.0 (20.3)	1.8 (0.40)	0.21 (0.07)	14.3 (2.9)
	PM (n=16)	3015422.9 (452807.3)	126074.0 (28528.9)	41.2 (13.3)	2.3 (1.1)	0.24 (0.06)	13.7 (2.4)
	p-value	0.165	0.164	0.381	0.525	0.117	0.264

Atorvastatin							
Gene	Genotype/phenotype	AUC/dW (ng·h·Kg/mL·mg)	C_{max}/dW(ng·kg/mL·mg)	$T_{1/2}$ (h)	T_{max}(h)	Cl (L/h·kg)	Vd (L/kg)
CYP3A4	Wild-type (n=44)	135931.4 (70279.7)	39656.5 (32807.8)	8.7 (2.5)	1.7 (1.2)	8.6 (2.8)	108.5 (49.7)
	Mutated (n=6)	115568.0 (33782.7)	22946.6 (7334.4)	8.5 (1.6)	1.2 (0.5)	9.2 (3.0)	114.1 (40.8)
	p-value	0.491	0.224	0.879	0.312	0.625	0.792
CYP3A5	NM (n=1)	167855.0	40372.5	8.0	0.7	5.9	67.6
	IM (n=5)	161978.3 (99720.8)	48787.7 (65431.8)	9.9 (3.3)	2.1 (2.2)	7.9 (4.1)	110.9 (55.1)
	PM (n=44)	129469.2 (63713.7)	36324.0(26499.6)	8.5 (2.3)	1.6 (1.0)	8.8 (2.7)	109.9 (48.4)
	p-value	0.525	0.706	0.465	0.514	0.483	0.694

Fentanyl							
Gene	Genotype/phenotype	AUC/dW (pg·h·Kg/mL·mg)	C_{max}/dW(pg·Kg/mL·mg)	$T_{1/2}$ (h)	T_{max}(h)	Cl (L/h·kg)	Vd (L/kg)
CYP3A4	Wild-type (n=33)	736.7 (377.2)	142.7 (44.6)	13.5 (6.0)	1.0 (0.4)	15.3 (0.8)	25.0 (11.6)

	Mutated (n=2)	1098.9 (87.9)	181.8 (14.4)	15.0 (3.1)	1.1 (0.2)	7.7 (1.0)	16.5 (1.3)
	p-value	0.190	0.232	0.728	0.756	0.216	0.314
CYP3A5	IM (n=5)	659.0 (293.2)	134.8 (40.2)	12.4 (8.7)	1.0 (0.3)	16.8 (10.2)	20.8 (6.0)
	PM (n=44)	773.8 (389.9)	146.7 (45.4)	13.7 (5.4)	1.0 (0.4)	14.5 (8.1)	25.2 (12.1)
	p-value	0.535	0.588	0.641	0.676	0.575	0.440

Fesoterodine

Gene	Genotype/phenotype	AUC/dW (ng·h·Kg/mL·mg)	C_{max}/dW (ng·Kg/mL·mg)	$T_{1/2}$ (h)	T_{max}(h)	Cl (L/h·kg)	Vd (L/kg)
CYP3A4	Wild-type (n=12)	608.6 (225.9)	60.6 (18.0)	8.3 (2.4)	5.6 (0.7)	1.9 (0.8)	23.0 (14.3)
	Mutated (n=1)	298.3	30.4	7.1	6.0	3.3	34.5
	p-value	0.214	0.135	0.650	0.637	0.119	0.457
CYP3A5	PM (n=13)	584.8 (232.8)	58.3 (19.1)	8.2 (2.3)	5.6 (0.7)	2.0 (0.9)	23.9 (14.0)

Imatinib

Gene	Genotype/phenotype	AUC/dW (ng·h·Kg/mL·mg)	C_{max}/dW(ng·kg/mL·mg)	$T_{1/2}$ (h)	T_{max}(h)	Cl (L/h·kg)	Vd (L/kg)
CYP3A4	Wild-type (n=11)	6077.5 (1303.6)	392.0 (79.7)	12.3 (1.7)	3.4 (1.3)	0.17 (0.04)	2.9 (0.5)
	Mutated (n=1)	9183.9	555.8	16.0	1.5	0.10	2.4
	p-value	0.046	0.077	0.072	0.196	0.148	0.321
CYP3A5	IM (n=1)	8178.3	438.0	16.1	3.0	0.11	2.7
	PM (n=11)	6168.9 (1487.9)	402.7 (93.3)	12.3 (1.7)	3.22 (1.4)	0.17 (0.04)	2.9 (0.5)
	p-value	0.225	0.724	0.060	0.880	0.271	0.753

Olanzapine

Gene	Genotype/phenotype	AUC/dW (pg·h·Kg/mL·mg)	C_{max}/dW (pg·Kg/mL·mg)	$T_{1/2}$ (h)	T_{max}(h)	Cl (L/h·kg)	Vd (L/kg)
CYP3A4	Wild-type (n=25)	2974478.5 (569795.8)	103677.2 (26770.0)	32.3 (9.3)	4.4 (2.3)	0.27 (0.06)	859.2 (207.2)
CYP3A5	NM (n=1)	2322306.8	107603.6	23.9	2.5	0.38	13.0
	IM (n=12)	3022146.2 (499342.1)	101261.4 (24583.9)	32.3 (6.9)	4.4 (1.9)	0.26 (0.05)	12.0 (2.1)

	PM (n=12)	2981158.5 (646396.8)	105765.9 (30767.9)	33.0 (11.7)	4.6 (2.7)	0.27 (0.07)	12.5 (3.1)
	p-value	0.517	0.915	0.668	0.704	0.248	0.880

Quetiapine							
Gene	Genotype/phenotype	AUC/dW (ng·h·Kg/mL·mg)	C_{max}/dW$_{(ng·kg/mL·mg)}$	$T_{1/2}$ (h)	T_{max}(h)	Cl (L/h·kg)	Vd (L/kg)
CYP3A4	Wild-type (n=17)	588.9 (272.6)	199.4 (106.3)	4.8 (1.1)	1.2 (1.1)	2.1 (1.1)	14.2 (8.6)
	Mutated (n=2)	1107.7 (376.1)	334.1 (92.0)	4.5 (0.8)	2.2 (2.5)	0.9 (0.3)	6.2 (3.1)
	p-value	0.024	0.106	0.668	0.305	0.173	0.218
CYP3A5	IM (n=6)	548.5 (211.0)	203.9 (78.0)	4.2 (1.5)	0.7 (0.2)	2.1 (1.1)	11.3 (3.4)
	PM (n=13)	687.4 (354.7)	218.0 (126.0=	5.1 (0.8)	1.6 (1.4)	1.9 (1.2)	14.4 (10.0)
	p-value	0.390	0.806	0.112	0.135	0.737	0.482

Abbreviation: AUC, area under the curve; C_{max}, maximum plasma concentration; T_{max}, time to reach the maximum plasma concentration; $T_{1/2}$, half-life; Cl, total drug clearance adjusted for bioavailability; Vd, volume of distribution adjusted for bioavailability; CYP, cytochrome p450 oxidase; dW, corrected by dose/weight.
